# Impact of *E. muris* infection on *B. burgdorferi–*induced joint pathology in mice

**DOI:** 10.3389/fimmu.2024.1430419

**Published:** 2024-08-20

**Authors:** Jesse L. Bonin, Steven R. Torres, Ashley L. Marcinkiewicz, Gerald E. Duhamel, Xiuli Yang, Utpal Pal, Julia M. DiSpirito, Tristan A. Nowak, Yi-Pin Lin, Katherine C. MacNamara

**Affiliations:** ^1^ Department of Immunology and Microbial Disease, Albany Medical College, Albany, NY, United States; ^2^ Division of Infectious Disease, Wadsworth Center, New York State Department of Health, Albany, NY, United States; ^3^ New York State Animal Health Diagnostic Center and Department of Biomedical Sciences, College of Veterinary Medicine, Cornell University, Ithaca, NY, United States; ^4^ Department of Veterinary Medicine, Virginia-Maryland Regional College of Veterinary Medicine, University of Maryland, College Park, MD, United States; ^5^ Department of Biomedical Sciences, State University of New York at Albany, Albany, NY, United States

**Keywords:** infection, *Borrelia* (*Borreliella*) *burgdorferi*, hematopoiesis, inflammation, *Ehrlichia*

## Abstract

Tick-borne infections are increasing in the United States and around the world. The most common tick-borne disease in the United States is Lyme disease caused by infection with the spirochete *Borrelia burgdorferi* (*Bb*), and pathogenesis varies from subclinical to severe. *Bb* infection is transmitted by *Ixodes* ticks, which can carry multiple other microbial pathogens, including *Ehrlichia* species. To address how the simultaneous inoculation of a distinct pathogen impacted the course of *Bb*-induced disease, we used C57BL/6 (B6) mice which are susceptible to *Bb* infection but develop only mild joint pathology. While infection of B6 mice with *Bb* alone resulted in minimal inflammatory responses, mice co-infected with both *Bb* and the obligate intracellular pathogen *Ehrlichia muris* (*Em*) displayed hematologic changes, inflammatory cytokine production, and emergency myelopoiesis similar to what was observed in mice infected only with *Em*. Moreover, infection of B6 mice with *Bb* alone resulted in no detectable joint inflammation, whereas mice co-infected with both *Em* and *Bb* exhibited significant inflammation of the ankle joint. Our findings support the concept that co-infection with *Ehrlichia* can exacerbate inflammation, resulting in more severe *Bb*-induced disease.

## Introduction

Tick-borne illnesses, historically relegated to specific geographic areas, are increasing in prevalence and epidemiological distribution. Ticks can carry multiple pathogens, and the geographical spreading of different tick species supports the idea that exposure to multiple tick-borne pathogens simultaneously is becoming increasingly more common. Whether transmission of multiple tick-borne pathogens simultaneously impacts disease outcomes and pathogenesis is largely unknown.

*Borrelia burgdorferi* sensu stricto (*Bb*), the causative agent of Lyme disease, is transmitted by *Ixodes* ticks, and is the most common tick-borne illness in North America (1–3). Infection with *Bb* often results in a characteristic skin rash called erythema migrans, followed by dissemination of the spirochete bacterium to other organs and tissues, including joints. Disease can vary from mild to severe, and may include debilitating arthritis, carditis, and neurologic deficits, although it remains unclear why certain infected individuals develop severe disease while others are only mildly symptomatic. The bacterium itself does not produce toxins (4), but rather the recruitment of immune cells, notably neutrophils, is thought to cause significant inflammation of the joint, contributing to arthritis (5, 6). The immune response to *Bb* is driven by recognition of bacterial lipoproteins by host TLRs. *In vitro* infection of human PBMCs results in production of TNF, IL-6, IL-10, and IL-1β (7). Systemic effects of *Bb*, as exhibited by changes to circulating immune cells, are not observed in patients with Lyme arthritis, which may be a result of the pathogen’s ability to suppress or evade acute inflammatory responses (8).

Human monocytic ehrlichiosis (HME) is another common tick-borne disease that results from infection with the intracellular pathogen *Ehrlichia chaffeensis* and *E. muris* eauclarensis, carried by the *Amblyomma americanum* (9) and *I. scapularis* (10) ticks, respectively. HME causes profound hematologic disturbances, including reductions in circulating platelets (thrombocytopenia) and lymphocytes (lymphopenia) (11). *Ehrlichia muris* (*Em*) is closely related to *E. chaffeensis* (12) and causes disease in C57BL/6 mice similar to what is observed in human patients with HME (13). Whereas hematologic disturbances are characteristic of tick-borne ehrlichioisis, hematological changes are atypical with Lyme disease (14). Patients with borreliosis that also exhibited hematological abnormalities were found to have a co-infection (15). Moreover, concurrent Lyme borreliosis with babesiosis or ehrlichiosis results in a wider range of symptoms, such as more flu-like symptoms than those seen with Lyme borreliosis alone (14). In fact, in 39% of suspected tick-borne infections, multiple pathogens were present (14). These data support the notion that infection with multiple pathogens poses an increased risk of more severe disease.

Hematopoietic stem and progenitor cells (HSPCs) reside in the bone marrow at homeostasis where they give rise to all blood and immune cells in adult mammals (16, 17). Blood production can be altered during infection via a process called “emergency myelopoiesis”. Emergency myelopoiesis is driven by inflammatory factors that promote the rapid generation of immune cells necessary for combatting microbial challenges (18–20). *Em* infection elicits production of interferon gamma (IFNγ) and modulates the hematopoietic stem cell (HSC) niche contributing to profound anemia and thrombocytopenia observed upon infection (13, 21, 22). How such responses and hematologic changes to one tick-borne pathogen may impact the pathogenesis of Bb-induced disease is unknown.

To address the impact of *Em* infection on the pathogenesis of *Bb*-associated disease, we used C57BL/6 (B6) mice because they develop only mild *Bb*-induced joint inflammation and experience a mild immune response to the spirochete bacteria (23, 24). We observed minimal inflammation in B6 mice infected with *Bb* alone, whereas *Bb* and *Em* co-infection resulted in hematologic changes and inflammatory cytokine production resembling those of *Em* singly-infected B6 mice. *Em*-associated changes correlated with the development of severe arthritis in mice co-infected with *Bb*. *Em* infection elicited emergency myelopoiesis, reduced circulating lymphocytes, and caused profound thrombocytopenia. B6 mice, which only develop mild arthritis with *Bb* infection (25), exhibited emergency myelopoiesis and severe joint inflammation when co-infected with *Em*. Our work identifies infection-induced hematopoietic response and immune activation as important drivers of disease and pathology in *Bb* infection, potentially contributing to the clinical presentation in a subset of human patients with Lyme disease.

## Methods

### Mice

Female, 6–8-week-old C57BL/6 (B6NTac) mice were purchased from Taconic (Germantown, NY). All animal experiments were performed following approval by the IACUCs at AMC (ACUP 20-04004) and Wadsworth (Protocol docket numbers 16-431, 19-451, 22-451).

### Bacteria

*Em* stocks were generated from infected splenocytes as previously described (26). *Bb* strain B31-A3 was grown in Barbour-Stonner-Kelly (BSK)-II compete medium at 33°C (27). Prior to infection, cultured *B. burgdorferi* was analyzed with PCR to confirm the presence of all plasmids (28).

### Inoculation of mice

Mice were administered the same dose of *Em (*100,000 genomic copies) and *Bb* (100,000 cells) via i.p. injection of bacteria in sucrose-phosphate-glutamate (SPG) buffer as described previously for ehrlichial pathogens (22, 26) as well as *Bb* (5, 29, 30). Mice were sacrificed at 10 or 22 days post infection (dpi) and tissues collected.

### Quantification of bacterial burden

Mouse tissues for bacterial burdens were collected at days 10 and 22 post-inoculation (dpi). DNA was extracted from murine spleens using an EZNA Tissue DNA kit (Omega Bio-Tek, Georgia, USA). *Em* burden in infected mice was quantified using spleens via RT-qPCR using a Mastercycler ep *Realplex* (Eppendorf, Hamburg, Germany) (31) (**Supplementary Table 1**). To test for *Bb* burdens, DNA from mouse ears and bladder were extracted using an EZ-10 Genomic DNA kit (Biobasic, Markham, ON, Canada). Quantitative PCR was then performed to quantify *Bb* loads. Spirochete genomic equivalents were calculated using an ABI 7500 Real-Time PCR System (ThermoFisher Scientific) in conjunction with PowerUp SYBR Green Master Mix (ThermoFisher Scientific) based on amplification of the Lyme borreliae *recA* gene (**Supplementary Table 1**) with the amplification cycle as described previously (32). The number of *recA* copies was calculated by establishing a threshold cycle standard curve of a known number of recA gene extracted from cultured B31-A3.

### Xenodiagnosis

Mice were infected as described above, and xenodiagnoses assays were initiated at 22 dpi. Single mice were restrained for 1 hour in a 50 mL conical tube containing approximately 100-150 naïve, unfed *Ixodes scapularis* larvae to allow attachment. Mice were then removed and placed in their home cage, located within a larger water moat. Mice can’t remove ticks located on their head, ears, and back of the neck, and actively feeding ticks are embedded in the skin and not easily removed. Larvae fed to repletion (3-5 days) and then dropped from the mice and were collected. Typical recovery was approximately 50-75 ticks per mouse. Collected ticks were frozen at -20°C until processing for analysis. Frozen ticks were crushed, and DNA was extracted using an EZ-10 Genomic DNA kit. Quantitative PCR was performed on ten ticks per individual mouse, at a minimum of two technical replicates. Individual ticks with an average *recA* burden above the lowest standard were considered positive, and mice with a minimum of one positive tick were considered to be infected.

### Blood collection for complete blood count and serum analysis

Blood was collected from mice via cardiac puncture into BD Microtainer Tubes with K_2_EDTA (Cat. No. 365974). CBC analysis was performed using a Heska ElementHT5 Veterinary Hematology Analyzer (Heska Corporation, Loveland, Colorado). For serum analysis, blood was collected via cardiac puncture, spun to pellet cells, and sera was removed for protein and chemokine analysis. The levels of total anti-*Bb* IgG in the sera were quantified as previously described (33).

### Protein analysis

Protein from bone marrow was collected by homogenization of bone marrow with a pestle in lysis buffer made with the detergent IGEPAL CA-630 (Octylphenoxy poly(ethyleneoxy)ethanol, branched; Sigma-Aldrich, St. Louis, MO) and Protease Inhibitor Cocktail (containing aprotinin, bestatin, E-64, leupeptin, and pepstatin A; P1860; Sigma-Aldrich, St. Louis, MO). Analysis of chemokines present in the serum and bone marrow of mice was performed using a Bio-Plex Pro Mouse Chemokine Panel 31-Plex (Bio-Rad, Hercules, California), and for bone marrow, data were normalized to total protein as quantified by BCA Protein Assay (Thermo Scientific, Waltham, MA).

### Flow cytometry

BM cells were isolated from murine tibia and femora via flushing with HBSS, followed by passage through a 70 μm sterile filter, and spleens were processed through crushing between frosted glass slides and passage through a 70 μm filter. Single-cell suspensions underwent red blood cell lysis via ACK buffer, followed by counting and plating. Cells were stained with fluorescently labeled antibodies described in **Supplementary Table 1**. Data were collected on a FACS LSR II or FACS Symphony (BD Biosciences, San Jose, California). Data were analyzed using FlowJo v10 (BD, San Jose, California).

### Histopathology

Tibiotarsal (ankle) joints were collected from mice at 22 dpi. Joints were fixed for 48 hours in 10% neutral-buffered formalin followed by decalcification with 10% formic acid, paraffin wax embedding, sectioning, and hematoxylin and eosin staining (Wadsworth Histopathology Core Facility, NYS Department of Health, Albany, NY, USA). Arthritis severity was scored as previously described (33, 34). Briefly, two sections per mouse joint were blindly evaluated for the severity of *Bb*-induced arthritis based on the inflammatory scores of 0 (no inflammation), 1 (mild inflammation with less than two small foci of infiltration), 2 (moderate inflammation with two or more foci of infiltration), or 3 (severe inflammation with focal and diffuse infiltration covering a large area). The inflammatory phenotypes include the infiltration of different cell types (i.e., neutrophils and histocytes), the swelling and edema of tendon sheaths, and bone remodeling. These phenotypes were only observed in the joints of co-infected mice.

### Statistical analysis

Statistical tests between multiple groups were performed using one-way ANOVA with Tukey’s multiple comparisons or Kruskal-Wallis non-parametric test. Statistical tests for xenodiagnostic ticks were performed using two-way Fisher’s exact test. All data were analyzed using GraphPad Prism 9 (San Diego, California).

## Results

### *E. muris*-induced cytopenias exacerbated by co-infection in B6 mice

**Figure 1:**
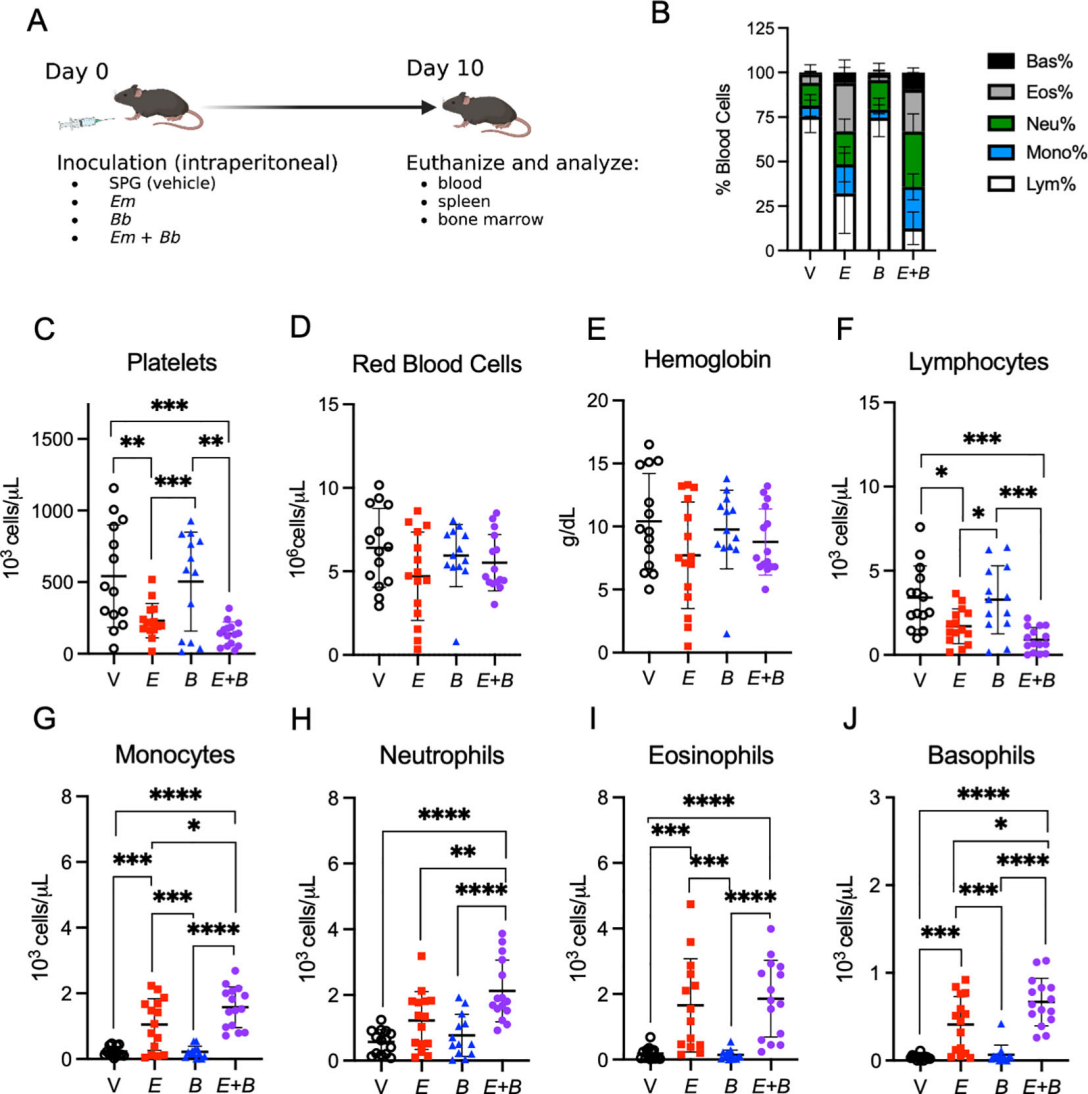
Co-infection of *E. muris* with *B. burgdorferi* exacerbate cytopenias. **(A)** B6 mice were inoculated with Vehicle (V, SPG), *E. muris* (*Em*), *B. burgdorferi* (*Bb*), or both *Em* and *Bb.* Mice were euthanized at 10-days post infection for analysis of tissues. Panel created with BioRender.com. Blood was collected and analyzed with a Heska HT5 CBC analyzer. **(B)** Composition of total white blood cells (WBC) is shown for V, *Em*, *Bb*, or *Em* and *Bb* co-infected mice including percent of lymphocytes, monocytes, and granulocytes (neutrophils, basophils, and eosinophils). **(C)** Platelets **(D)** red blood cells and **(E)** hemoglobin are shown for all groups. Total numbers of circulating **(F)** lymphocytes, **(G)** monocytes, **(H)** neutrophils, **(I)** eosinophils, and **(J)** basophils are shown. Data points represent individual mice; error bars represent standard deviation. Groups were compared using a one-way ANOVA with Tukey’s *post-hoc* comparison. *p<0.05, **P<0.01, ***p<0.001, ****p<0.0001. Data are pooled from 3 experiments, n=14-15 mice per group.

C57BL/6 (B6) mice can be colonized by *Bb* but develop only mild Lyme borreliosis-associated disease manifestations (e.g. arthritis) compared with other mouse strains (25, 35). To determine the impact of co-infection on pathogenesis of *Bb*-induced disease, B6 mice were inoculated singly or simultaneously via i.p. injection with *Bb* and *Em* (**Figure 1A**). Although not a physiological route of inoculation, the i.p. route of delivery has been extensively used in the study of *Em* infection and has been shown to result in transmission of *Bb* in mice (22, 36–40). This experimental set-up allowed simultaneous delivery of both pathogens, which was not feasible using tick transmission or intradermal inoculation.

Ehrlichiosis is accompanied by severe hematologic disturbances (22); therefore, we first investigated hematologic parameters of infected mice at the peak of *Em* infection [10 dpi (21)] (**Figure 1A**). As expected, complete blood count (CBC) data revealed *Em* infection induced profound lymphopenia, an increased proportion of myeloid cells, and severe thrombocytopenia, whereas *Bb* infection resulted in no obvious hematologic changes (**Figures 1B, C**). Co-infected mice exhibited similar hematologic changes as single *Em* infection, although thrombocytopenia appeared somewhat more severe in mice inoculated with both *Em* and *Bb*, compared to those singly infected with *Em* (**Figure 1C**) Mild anemia was seen in all infected mice (**Figures 1D, E**). Mice infected with *Em* had reduced circulating lymphocytes, which was slightly more pronounced in the group infected with both *Em* and *Bb* (**Figure 1F**).

Monocytes were significantly increased upon *Em* infection at 10 dpi, while *Bb* infection alone caused no perturbation in myeloid cells, and closely resembled vehicle-inoculated controls (**Figure 1G**). Mice infected with both Em and Bb had significantly increased circulating monocytes relative to *Em* infection alone. Similarly, we observed significantly increased neutrophils and basophils in mice infected with both *Em* and *Bb*, relative to *Em* infection (**Figures 1H–J**). These data suggest that infection-induced changes in hematological parameters were primarily driven by *Em*, though mice infected with both pathogens exhibited significantly elevated neutrophils, monocytes, and basophils and relative to singly *Em*-infected mice. Therefore, we conclude that the presence of *Em* dominated the early innate immune response, but co-infection increased the degree of hematologic changes.

To begin to characterize the systemic immune response in mice inoculated with both pathogens, we quantified cytokines and chemokines in the serum. In co-infected mice the inflammatory cytokines IL-1β, IL-6, IL-16, TNF, and interferon gamma (IFN-γ) were all elevated (**Supplementary Figure 1A**). IFN-γ was previously found to be an important driver of *Em*-induced myelopoiesis (13) and was elevated in the context of both single *Em* and co-infection, although it remained similar to vehicle controls during single *Bb* infection. Increased CXCL10 was only observed in mice co-infected with both *Em* and *Bb* (**Supplementary Figure 1B**). The chemokine CXCL12, which retains HSPCs in the bone marrow, was significantly decreased in *Em*-infected mice. We observed that concentrations of the monocyte chemoattractants CCL2, CCL3, CCL4, CCL5, and CCL7 were significantly increased in mice inoculated with both pathogens, relative to vehicle and single *Bb* infection (**Supplementary Figure 1C**). The chemokine CCL4 was also significantly increased in co-infected mice, relative to *Em* alone. Together, these data illustrate a systemic inflammatory response elicited by *Em* infection that may modulate blood cell production and migration in the context of co-infection with *Bb*.

To investigate bacterial growth in mice, spleens were obtained to determine ehrlichial burdens by quantitative PCR (21). *Em* was detected in single and co-infected mice at 10 dpi with similar burdens among the two groups (**Supplementary Figure 2A**). PCR was also used to determine *Bb* burdens in multiple tissues (i.e., ears and bladder) at day 10, which is an early time point for borreliosis (6). We failed to detect any burdens (**Supplementary Figure 2B**), which may be due to the i.p. inoculation, which can be less efficient relative to other routes of infection (i.e., i.d. or tick feeding) (36), in addition to the fact that B6 mice are less susceptible to *Bb* infection (41). We thus examined sera at days 10 and day 22 dpi to evaluate *Bb*-specific antibody responses to demonstrate that inoculation of Bb resulted in infection and an immune response. Anti-*Bb* IgG antibodies were not detectable at 10 dpi (**Supplementary Figure 2C**), as expected; however, titers were present in *Bb*-infected mice at 22 dpi (42), with no difference between those inoculated with *Bb* alone or in conjunction with *Em* (**Supplementary Figure 2D**). These data suggest that both *Em* and *Bb* were able to establish infection in mice via the i.p. route. Inoculation of mice with *Bb* did not appear to impact *Em* burdens, and, at the same time, inoculation of mice with *Em* and *Bb* did not impact antibody responses directed at *Bb*.

### *E. muris* drives increased myelopoiesis

**Figure 2:**
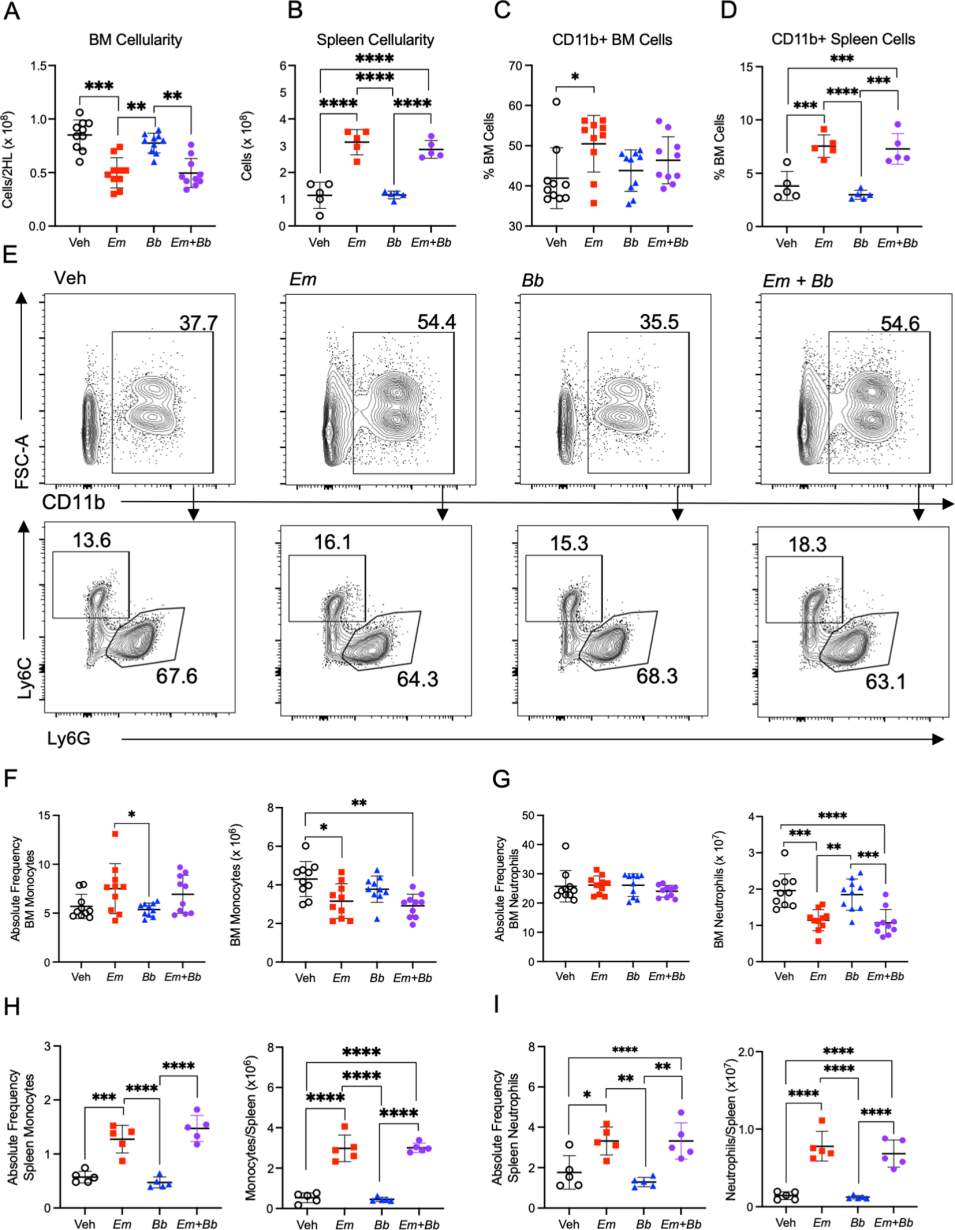
Co-infection impacts mature myeloid cells in the bone marrow and spleen at 10 dpi. B6 mice were inoculated with Vehicle (Veh), *E muris* (*Em*), *B burgdorferi* (*Bb*), or both *Em* and *Bb.* Hind limbs and spleens were harvested from single- and co-infected mice 10 days post infection. Total numbers of **(A)** bone marrow and **(B)** spleen cells were quantified. CD11b^+^ cells in the **(C)** bone marrow and **(D)** spleen. **(E)** Gating strategy to identify CD11b^+^ cells (top row) and Ly6C^+^ monocytes and Ly6G^+^ neutrophils cells within the CD11b^+^ population in the bone marrow of inoculated animals (bottom row). The numbers indicate frequencies of gated region among CD11b^+^ cells. **(F)** CD11b^+^ Ly6G^-^ Ly6C^+^ monocytes and **(G)** CD11b^+^ Ly6G^+^ Ly6C^lo^ neutrophils were quantified in the bone marrow. Splenic **(H)** monocytes and **(I)** neutrophils were quantified in the same manner. Data points represent individual mice. Cell numbers were compared using a one-way ANOVA with Tukey’s *post-hoc* comparison. Error bars represent standard deviation. *p<0.05, **p<0.01, ***p<0.001, ****p<0.0001. Data are pooled from 2 experiments, n=5-10 mice per group.

Given the significant changes in the composition of the blood, we next sought to evaluate the major site of immune cell development. Bone marrow cellularity of *Em*-infected mice was significantly reduced compared to control animals, consistent with previous findings (22); bone marrow was similarly hypocellular in mice inoculated with both pathogens whereas infection with *Bb* alone induced no overt changes (**Figure 2A**). *Em* infection causes significant splenomegaly (13, 22), which was also observed in mice infected with both *Em* and *Bb* (**Figure 2B**). The population of CD11b+ myeloid cells underwent significant expansion in the bone marrow and spleens of *Em* and co-infected animals (**Figures 2C, D**). We further characterized CD11b+ immune cells based on Ly6G and Ly6C expression to identify monocytes (Ly6C^hi^ Ly6G^-^), and neutrophils (Ly6C^lo/-^ Ly6G^+^) (**Figure 2E**). Despite the increased frequency of CD11b+ cells, absolute numbers of monocytes and neutrophils were reduced in the bone marrow of both *Em* and co-infected animals (**Figures 2F, G**), and no changes to these mature cell populations was noted in mice infected with *Bb* alone (**Figures 2H, I**). While reduced in the bone marrow, both monocytes and neutrophils were significantly increased in the spleen in *Em*-infected mice and mice co-infected with both *Em* and *Bb*. The simultaneous decrease in bone marrow myeloid cells and increase in blood and spleen myeloid cells is consistent with the observation that *Em* infection elicits rapid production and mobilization of myeloid cells (13). Our observations demonstrate that *Em*-driven myelopoiesis can also occur in the context of *Bb* infection.

**Figure 3:**
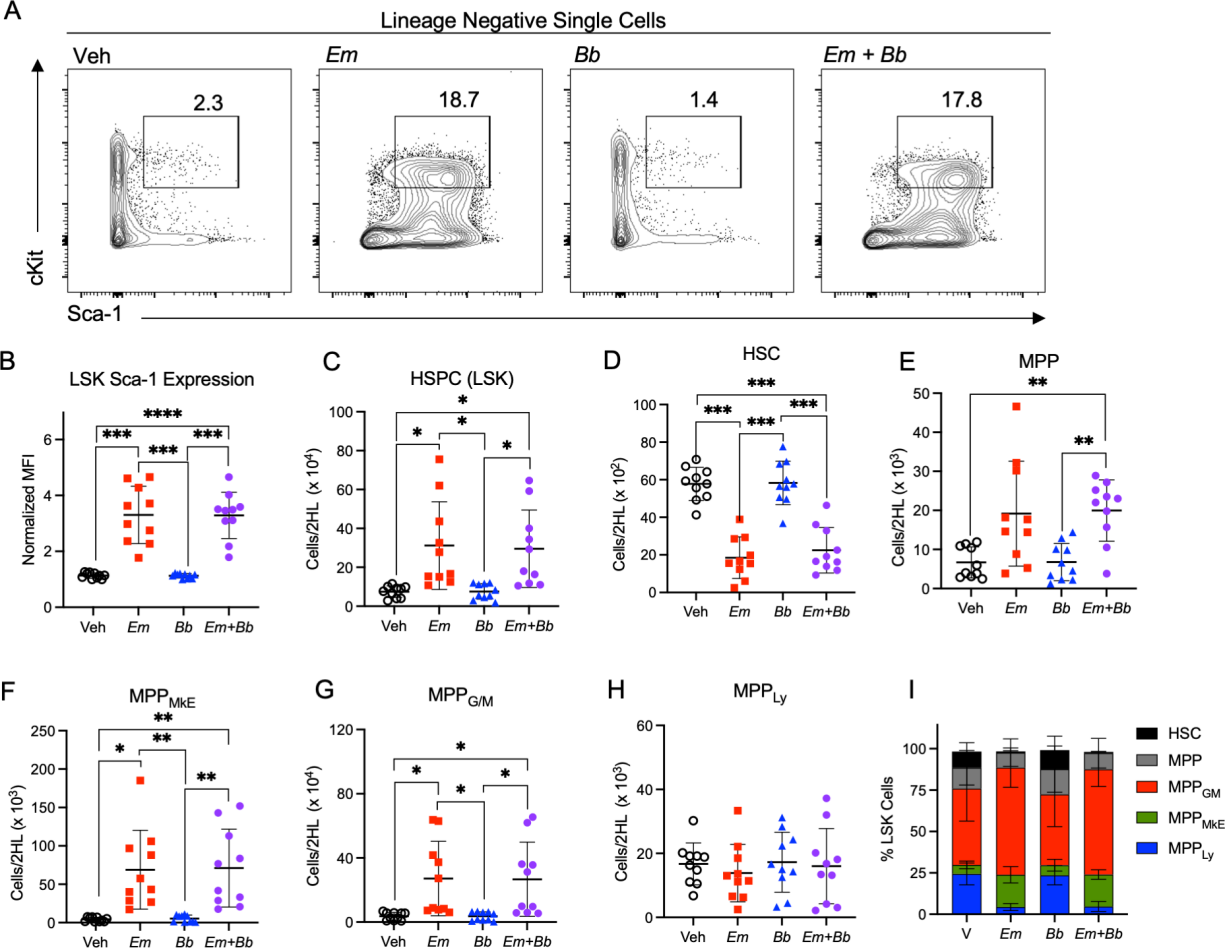
Co-infection drives loss of myeloid-biased progenitor cells in the bone marrow. B6 mice were inoculated with Vehicle (Veh), *E. muris* (*Em*), *B. burgdorferi* (*Bb*), or both *Em* and *Bb.* Bone marrow was isolated and stained to identify hematopoietic stem and progenitor cells at 10 days post infection. **(A)** Gating shows cKit and Sca-1 expression on Lineage-negative cells and the gated region reflects the percent of Sca-1^+^ cKit^+^ cells among the parent Lin^-^ gate. **(B)** Sca-1 expression on LSK cells is shown. **(C)** Total Lin^-^ Sca-1^+^ cKit^+^ (LSK) HSPCs are shown for Vehicle (SPG) controls and mice infected with *Em, Bb* or co-infected both pathogens. Hematopoietic stem cells (HSC) and multipotent progenitors (MPPs) were gated based on expression of CD135, CD150, and CD48 and the absolute numbers of **(D)** HSCs, **(E)** MPPs, **(F)** MPP_MkE_, **(G)** MPP_G/M_, and **(H)** MPP_Ly_ are shown. **(I)** The distribution of progenitor cells within the LSK population is shown. Data points represent individual mice. Groups were compared using a one-way ANOVA with Tukey’s *post-hoc* comparison. Error bars indicate standard deviation. *p<0.05, **p<0.01, ***p<0.001, ****p<0.0001. Data are pooled from 2 experiments n=10 mice per group.

To determine whether changes in the hematologic parameters of infected mice correlated with changes within the hematopoietic stem and progenitor cell (HSPC) compartment, mice were sacrificed at 10 dpi, and bone marrow was processed for flow cytometric analysis (**Supplementary Figure 3**). First, we noted that Lineage-negative cells exhibited significantly increased Sca-1 expression in mice singly- or co-infected with *Em* (**Figures 3A, B**). The total pool of Lineage^-^ cKit^+^ Sca-1^+^ (LSK) HSPCs was increased in the bone marrow of *Em* and co-infected mice (**Figure 3C**). Phenotypically-defined HSCs (LSK CD135^-^ CD150^+^ CD48^-^), which self-renew and give rise to more committed progenitor cells, were reduced in *Em* and co-infected animals, while short-term HSCs (also called multipotent progenitors, MPPs: LSK CD135^-^ CD150^-^ CD48^-^ (43);), were slightly increased (**Figures 3D, E**). Among the more lineage-committed HSPC populations, megakaryocyte-erythroid progenitors (MPP_MkE_) and myeloid-biased progenitors (MPP_G/M_) (43) were increased (**Figures 3F, G**), whereas lymphoid-biased progenitors (MPP_Ly_) were unchanged during infection (**Figure 3H**). Overall, the composition of those cells changed as well (**Figure 3I**) with an increase in myeloid-biased progenitors, which correlated with the infection-induced increase in circulating myeloid cells. There were no significant differences between single-*Em* and co-infected mice, suggesting that during co-infection the immune phenotype is dominated by responses elicited against *Em*.

### *Bb* infection prolongs and exacerbates *Em*-driven thrombocytopenia and monocytosis

**Figure 4:**
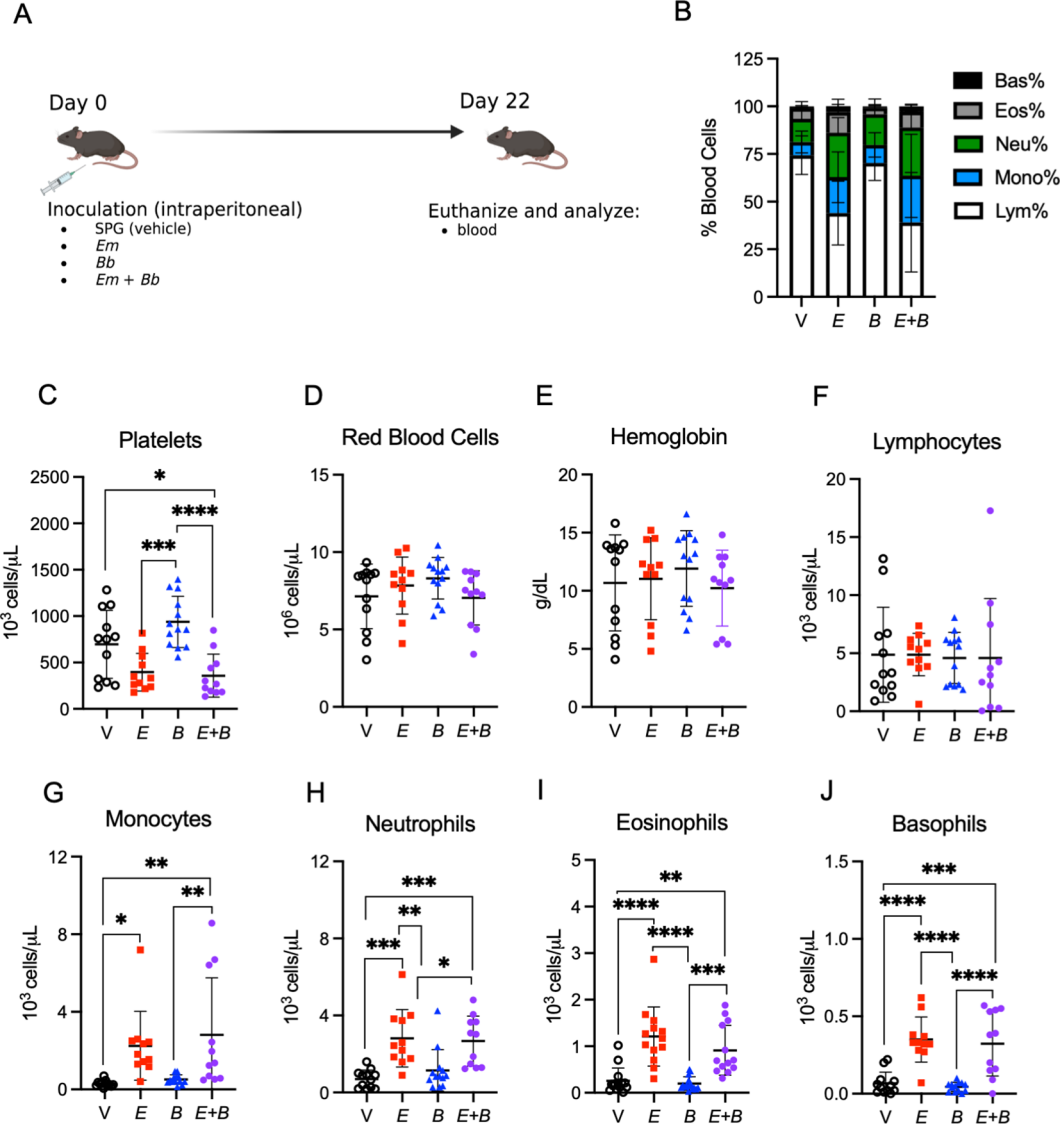
Myeloid cells remain elevated while lymphocytes return to normal in the blood. **(A)** B6 mice were inoculated with Vehicle (V, SPG), *E. muris* (*Em*), *B. burgdorferi* (*Bb*), or both *Em* and *Bb*, and euthanized at 22 dpi, the peak of *Bb* infection. **(B)** Composition of total white blood cells (WBC) is shown for V, *Em*, *Bb*, or *Em* and *Bb* co-infected mice including percent of lymphocytes, monocytes, and granulocytes (neutrophils, basophils, and eosinophils). **(C)** Platelets, **(D)** red blood cells, and **(E)** hemoglobin is shown for all groups. Total numbers of circulating **(F)** lymphocytes, **(G)** monocytes, **(H)** neutrophils, **(I)** eosinophils, and **(J)** basophils. Data represent individual mice. Groups were analyzed using a one-way ANOVA with Tukey’s *post-hoc* comparison. Error bars represent standard deviation. *p<0.05, **p<0.01, ***p<0.001, ****p<0.0001. Data are pooled from 2 experiments, n=11-12 mice per group.

We next sought to examine the hematological parameters at day 22 post-infection, the peak of *Bb* infection (6) (**Figure 4A**). Mice inoculated with *Em* alone, or in conjunction with *Bb*, displayed altered blood composition in comparison to healthy control mice and singly *Bb*-infected mice (**Figure 4B**). Mice infected with both *Em* and *Bb* exhibited the most significant degree of thrombocytopenia; however, normal numbers of red blood cells and hemoglobin were observed in all groups (**Figures 4C–E**) and *Em*-induced lymphopenia was resolved by day 22 (**Figure 4F**). Myeloid cells were elevated in *Em*-infected mice, though monocytosis was most pronounced in mice co-infected with both *Em* and *Bb* relative to uninfected mice (**Figures 4G–J**). Similar to observations at day 10, *Bb* inoculation did not induce changes to hematological parameters. Therefore, *Em* infection concurrent with *Bb* infection resulted in distinct hematological changes, as compared to single *Bb* infection, marked by elevated numbers of myeloid cells in the blood.

### Distinct chemokine and cytokine profiles in mice infected with both *Em* and *Bb*

**Figure 5:**
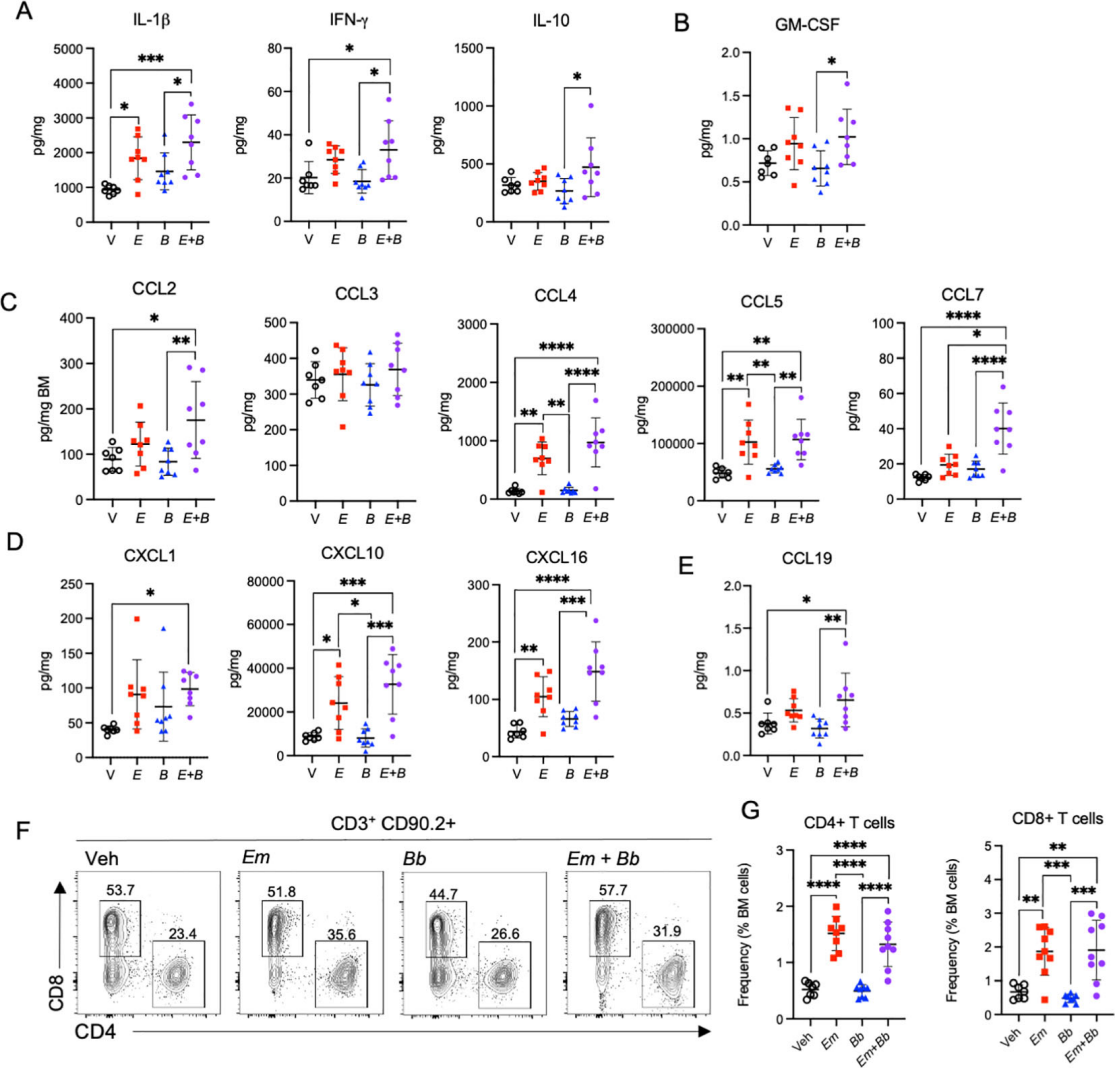
Co-infection drives persistent inflammation in the bone marrow. B6 mice were inoculated with Vehicle (V, SPG), *E. muris* (*Em*), *B. burgdorferi* (*Bb*), or both *Em* and *Bb*, and euthanized at 22 dpi. Bone marrow cells and protein were collected for flow cytometry and analysis of inflammatory cytokines and chemokines. **(A)** IL-1β, IFN-γ, and IL-10 are shown. **(B)** GM-CSF, **(C)** Monocyte chemoattractants CCL2, CCL3, CCL4, CCL5, CCL7 are shown. **(D)** CXCL1 and CXCL10, and **(E)** CCL19 are graphed. **(F)** Flow cytometric gating of CD90.2^+^ CD3^+^ lymphocytes for CD4+ or CD8+ T cells. The number above each gated region is the percent of parent gate for representative mice in each group. **(G)** Absolute frequencies of CD90.2^+^ CD3^+^ CD4^+^ T cell and CD90.2^+^ CD3^+^ CD8^+^ T cells among total bone marrow cells are shown. Data points represent individual mice. Groups were compared using a one-way ANOVA with Tukey’s *post-hoc* comparison. Error bars represent standard deviation. *p<0.05, **p<0.01, ***p<0.001, ***p<0.0001. Data are pooled from 2 experiments, n=7-8 mice per group.

To determine whether inoculation of *Bb* impacted *Em*-driven cytokine and chemokine responses in the hematopoietic compartment, we evaluated these proteins in the bone marrow at 22 dpi. The inflammatory cytokine IL-1β was significantly increased in *Em* infected mice in the context of both single and co-infection; however, the co-infected mice exhibited a more marked increase as compared to vehicle controls (**Figure 5A**). In addition, both IFN-γ and IL-10 were significantly elevated in mice co-infected with both *Em* and *Bb*, relative to *Bb* infection alone. The hematopoietic growth factor GM-CSF, which contributes to the growth of granulocytes and monocytes, was significantly elevated in mice infected with both *Em* and *Bb* relative to *Bb* alone (**Figure 5B**). The monocyte chemoattractants CCL2 and CCL4 were significantly increased in mice infected with both *Em* and *Bb*, relative to vehicle or single *Bb* infection, and CCL7 was significantly increased in co-infected mice relative to *Em* alone (**Figure 5C**). CCL5 was also significantly increased in *Em*-infected mice, although co-infection with *Bb* had no additional impact on levels of CCL5 in the bone marrow. The regulatory chemokines CXCL1 and CXCL16 were also increased by infection with *Em* though concentrations appeared to be increased more dramatically by co-infection (**Figure 5D**). CXCL10, which was increased in the sera at day 10 in *Em* and *Bb* co-infected mice was also significantly increased in the marrow of *Em* infected mice, including co-infected mice. Therefore, a unique inflammatory signature was observed in the bone marrow of co-infected mice, with markedly increased markers of inflammation compared to *Bb* infection alone. Moreover, specific chemokines exhibited a more pronounced increase than what was observed by single infection with *Em*.

The T cell chemokine CCL19 was also significantly elevated in the bone marrow in co-infected mice (**Figure 5E**). As CCL19 is a chemotactic signal for T cells we evaluated the lymphocyte compartment in the bone marrow. CD3^+^ CD90.2^+^ cells were gated on CD4 and CD8 expression to identify CD4+ and CD8+ T cells (**Figure 5F**). Significantly elevated frequencies of both CD4 and CD8 T cells were observed in the bone marrow of both *Em* and co-infected mice (**Figure 5G**), suggesting the *Em*-driven inflammatory phenotype seen in mice at 10 dpi persists to 22 dpi and promotes accumulation of lymphocytes in the bone marrow.

### Infection with both *Em* and *Bb* induces joint pathology in B6 mice

**Figure 6:**
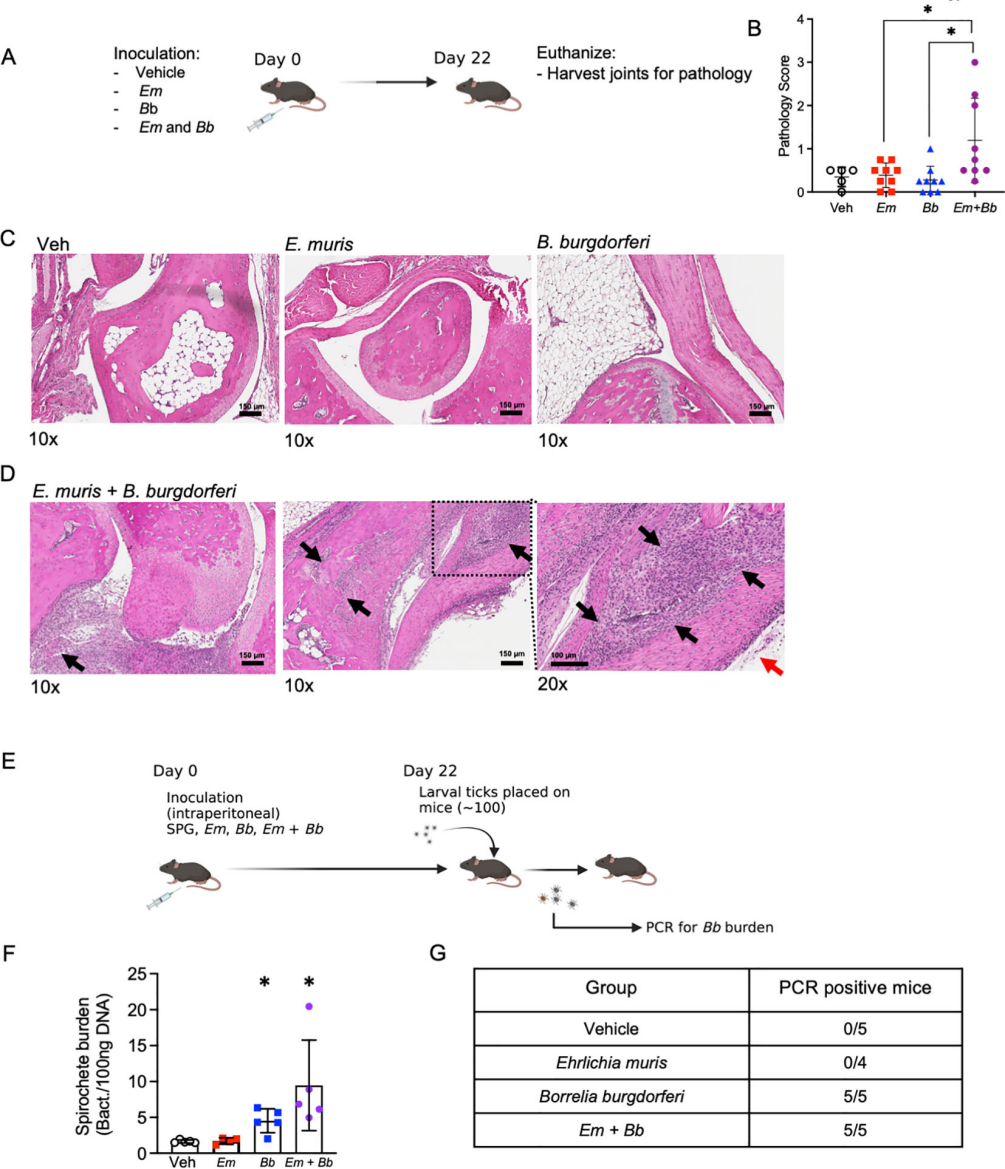
*Bb* and *Em* co-infected B6 mice develop severe tibiotarsal arthritis. **(A)** Mice were inoculated with vehicle, *Em*, *Bb*, or *Em* and *Bb*, and tibiotarsal (ankle) joints were collected at 22 dpi to assess inflammation by histological examination of hematoxylin and eosin stained formalin-fixed and paraffin-embedded tissue sections. **(B)** Quantitative analysis of joint pathology, as described in the Materials and Methods. **(C, D)** Representative photomicrographs of tibiotarsal joint taken from B6 mice in Veh, *Em*, and *Bb* group. Images of two co-infected mice are shown [scale bar, 150 µm]. Arrows indicate histopathological findings described in the result section. Magnification (10x or 20x) is shown below each panel. **(E)** To confirm infection with *Bb*, xenodiagnosis assays were performed at 22 dpi. Briefly, larval ticks were placed on mice, allowed to feed, and then analyzed for *Bb*. **(F)** Ticks were collected after feeding and PCR was performed to test for the presence of *Bb* genetic material. Average spirochete burden is shown for the pooled ticks from each individual mouse in each group. **(G)** Summary table of data from xenodiagnosis assays. Data points indicate individual mice, error bars represent median with 95% CI. Data were analyzed using a Kruskal-Wallis with multiple comparison, *p<0.05. Joint pathology data represent data pooled from 2 experiments, n=5-9 mice per group. Xenodiagnosis data are from one experiment n=4-5 mice per group.

We next assessed histomorphological changes in tibiotarsal (ankle) joints of infected mice by routine hematoxylin and eosin staining. At the day 10 time point, coinciding with the peak of *Em* infection (21), little to no inflammation was observed in the joints (data not shown). At 22 dpi, the respective peak for *Bb* infection (6), minimal changes were observed in the joints of mice singly infected with *Em* or *Bb* (**Figures 6A–C**). In contrast, mice co-infected with *Em* and *Bb* had tibiotarsal joints with proliferative synovium admixed with fibrin and inflammatory cells (left panel **Figure 6D**). Additionally, these mice developed localized periosteal bone remodeling of the distal tibia characterized by osteoclastic bony resorption and replacement by woven bone (middle panel **Figure 6D**). Higher magnification revealed severe, locally extensive swelling associated with edema and infiltration of the flexor and extensor tendon sheaths by large numbers of neutrophils and histiocytes (right panel **Figure 6D**).

The observation that single infection with *Bb* elicited little to no change in inflammation or pathology raised the possibility that inoculation with *Bb* alone did not establish productive or persistent infection in B6 mice. In addition, our data suggested that *Em* infection in conjunction with *Bb* may have caused persistence of *Bb*. To rigorously test whether *Bb* established infection in B6 mice, and to determine the impact of infection of *Em* on *Bb* persistence, we performed a xenodiagnosis assay. Xenodiagnosis is a robust and sensitive method to determine spirochete infection in mammals by allowing feeding of naïve larval ticks and subsequently testing for spirochete burdens in the fed ticks (**Figure 6E**) (44, 45). We allowed naïve larval ticks to begin feeding on mice at day 22 (**Figure 6F**) and found that mice infected with *Bb* alone or co-infected with both *Bb* and *Em* transmitted *Bb* to larval ticks (**Supplementary Figure 4**), demonstrating that all mice were actively infected with *Bb* (**Figure 6G**). Therefore, our observation that only co-infected mice developed disease was likely not due to failure of *Bb* to actively infect mice when inoculated in isolation, but rather due to differences in host immune responses and inflammation generation in co-infected animals. Together, our study supports the use of the B6 mouse model to understand host factors that contribute to joint pathology and *Bb*-induced disease. These data are also in line with the idea that disease outcomes may be significantly impacted by concurrent or subsequent infections, helping to explain the variation in disease trajectories seen in human patients infected with *Bb*.

## Discussion

Lyme disease, caused by *Bb*, is the most common tick-borne disease in the US (46, 47), and while a majority of patients treated with antibiotics recover fully from *Bb* infection, some patients exhibit persistent symptoms, such as arthritis, myalgia, and neurocognitive problems. B6 mice do not typically show significant joint pathology during *Bb* infection, whereas C3H mice develop both arthritis and carditis; thus, C3H mice have been used to study *Bb*-associated pathologies that can occur in human patients (41). In this study, while B6 mice exhibited few signs of *Bb* infection in singly infected mice, co-infection with *Em* resulted in a disease phenotype. While additional studies are required to delineate mechanisms driving the distinct pathological outcomes, the present work provides key proof-of-concept evidence that systemic inflammatory responses, elicited by other infectious organisms, can promote demand-adapted hematopoiesis and *Bb*-induced joint inflammation.

*Em* causes significant hematologic disturbances (48), and the distinct cytopenias and emergency myelopoietic responses observed in *Em* and *Bb* co-infected mice mirrored single *Em*-infected animals. Most notably we observed profound monocytosis at 10 dpi, the peak of *Em* infection (21), which persisted in co-infected mice at 22 dpi, the relative peak of *Bb* infection (6). The presence of neutrophils in the joint lesions supports the idea that granulocytes contribute to joint pathology. B6 mice infected with *Bb* alone, however, exhibited no detectable disturbances to their hematologic parameters, and resembled vehicle-treated mice with respect to joint pathology. It is a well-documented phenomenon that *Bb* evades or suppresses host immune responses during infection. For example, expression of CspA, CspZ, and OspE-related proteins act to inhibit the alternative pathway of complement activation to prevent cell lysis and inflammation (8). Thus, it is unlikely that *Bb* itself is sufficient for joint inflammation, but rather, host factors play a key role in determining disease progression and such factors can be altered by the co-infection of other tickborne pathogens. Previous studies demonstrate the difference in bacterial burden between an arthritis-resistant and arthritis-susceptible strain was negligible (48), further supporting our conclusion that factors within the infected host impact disease outcome. The hematologic parameters observed in murine co-infection were consistent with those seen in human co-infection with *Bb* and *Ehrlichia* species (14), further supporting the clinical relevance of our findings. Determining the mechanisms that result in recruitment to the joint tissue in co-infected animals will be critical for treating joint pathology in Lyme disease patients.

Crandell et al. demonstrated that C57BL/6 mice have reduced expression of IFN-induced genes and elevated expression of genes associated with tissue repair when compared to C3H mice (49), which has been postulated to account for resistance of C57BL/6 mice in developing severe joint pathology. Our data suggest resistance to *Bb*-induced joint pathology can be overcome by inoculation with a co-infecting pathogen that elicits IFN-γ. In support of this conclusion, it has been established that IFN-γ is critical for control of *Em* infection (22, 50); thus, inflammatory responses seen in co-infected mice are likely an adaptation of the host to control *Em* infection. Infection with *Em* was shown to result in transient IFN-γ-dependent activation of HSCs, which was also associated with production of monocytes and granulocytes (13). Consistent with Crandall et al. (49), B6 mice infected with *Bb* alone did not produce IFN-γ, and did not develop joint inflammation. However, during co-infection with both *Bb* and *Em*, B6 mice produced high levels of IFN-γ and experienced profound infiltration of neutrophils and lymphocytes into the ankle joint. Together, these data suggest that the presence of *Em*-elicited IFN-γ during *Bb* infection of B6 mice may be one potential mechanism that contributed to joint pathology in otherwise resistant mice.

Despite the observation that *Bb* is not a strong inducer of IFNγ (51), elevated IFNγ has been associated with *Bb*-induced disease in humans in various contexts. In human patients where antibiotic treatment failed to treat Lyme arthritis, gene expression analysis identified a robust IFNγ signature in the inflamed joints (52). At the same time, IFN-γ was identified in the blood and cerebrospinal fluid of infected individuals, and high levels of IFN-γ were associated with long-lasting inflammation and antibiotic-refractory arthritis (53, 54). Moreover, the combination of *Bb* and exogenous IFN-γ resulted in synergistic production of chemokines from human endothelial cells, relative to *Bb* alone (55). Therefore, while *Bb* may not elicit robust production of IFN-γ, persistent inflammatory sequelae appear to correlate significantly with IFN-γ. Consistent with our findings, co-infection of mice with *Bb* and *Babesia microti* resulted in alterations to the immune response against both pathogens, with more severe arthritis and lower *Babesia microti* burdens in co-infected mice (56, 57). The pronounced IFN-γ signature in the bone marrow of co-infected mice suggests that IFN-γ-induced hematopoietic programs persist and produce cells that may contribute to joint inflammation.

In support of a role for IFN-γ in promoting pathology, we noted that co-infected mice exhibited significantly elevated levels of IFN-γ-induced chemokines within the bone marrow at day 22. CCL2 and CCL7 are important signals for mobilization of monocytes and recruitment of monocytes to sites of inflammation (58, 59), and as monocyte-lineage cells promote inflammatory hematopoietic programs (60) these chemokines may contribute to persistent hematologic sequelae in co-infected mice. CXCL10 has also been noted to be elevated in the cerebrospinal fluid of patients with neuroborreliosis (61). CXCL10 and CXCL16 are potent monocyte, macrophage and T cell recruiting chemokines. In addition, elevated CCL19, specifically in co-infected animals, correlated with the increase in T cells in the bone marrow. Therefore, the direct mediators of increased pathology in the context of co-infection likely includes chemotactic signals that promote recruitment of inflammatory cells to the bone marrow, where they maintain signals to promote myeloid cell production. T lymphocytes drive hematopoietic activity in the context of various viral and bacterial infections (37, 62), implicating their accumulation in pathology observed in co-infected mice.

An important consideration of this work is that infectious organisms were introduced via needle inoculation to the peritoneal cavity. In physiological settings *Bb* and ehrlichial pathogens are transmitted to a host via tick feeding on the skin. Tick bites transmit not only bacteria but also salivary proteins and tick salivary proteins aid in establishment of infection through suppression of local host immune mechanisms (63). The *Ixodes scapularis* protein Salp15 has been shown to protect *B. burgdorferi* from antibody-mediated killing in a murine model of Lyme disease (64), and it serves as an anticoagulant that promotes the length of tick feeding (65). Sertour et al. found that significant changes in tissue tropism occurred between mice that were infected via needle inoculation and tick feeding (36); therefore, it stands to reason that the i.p. route of pathogen administration used here may play a role in the resulting immune response. However, tick feeding itself results in a localized host immune response, which will introduce additional variables that may impact disease outcomes (65), and therefore, future work is needed to evaluate specific impacts of tick feeding on bacterial persistence and arthritis development in the context of co-infection.

The observation that co-infection elicited more pronounced inflammatory changes during early stages of co-infection suggested an early interaction between the host and co-infecting pathogens that created a permissive environment for the development of arthritis. The tropism of the spirochete for joints and the increased presence of inflammatory cells in the blood of co-infected mice may have created a scenario that enabled neutrophils and monocytes to home to the joint, driving arthritis. Furthermore, we made the novel observation that emergency myelopoiesis programs correlated with progression of joint arthritis. Considering recent observations that inflammation-induced hematopoietic priming can worsen osteoarthritis (66), the impact of co-infection on blood cell production may be crucial for joint pathology. These studies provide new insight to how tick-borne infections may interact in the mammalian host to elicit unique inflammatory profiles that activate hematopoietic programs and contribute to disease progression. Further studies are needed to identify the cell types and signaling molecules necessary to initiate and perpetuate disease in the B6 co-infection model of *Bb*-induced joint pathology.

## Data Availability

The raw data supporting the conclusions of this article will be made available by the authors, without undue reservation.
