# Supplementary material for: Impact of E. muris infection on B. burgdorferi–induced joint pathology in mice
Source: Front Immunol. 2024 Aug 20;15:1430419. doi: 10.3389/fimmu.2024.1430419 (PMC11368855; doi:10.3389/fimmu.2024.1430419)
Supplement: Supplementary file 1 [file Datasheet1.pdf]

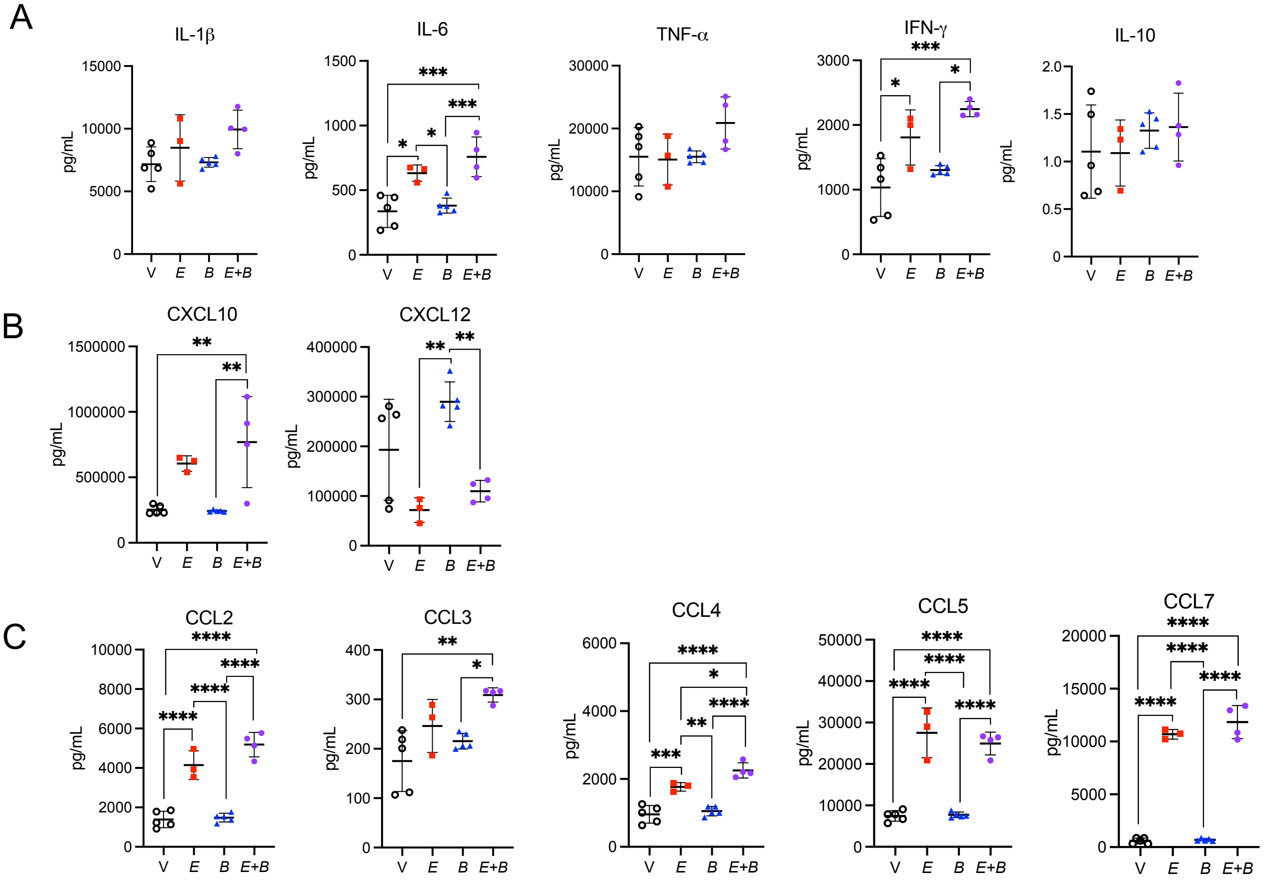

Supplemental Figure 1. Circulating cytokines in single and co-infected mice. B6 mice were inoculated with Vehicle (V), *Em (E)*, *Bb (B)*, or both pathogens (*E*+*B*). At 10 dpi, serum was collected and analyzed by multiplex. Inflammatory factors associated with emergency myelopoiesis and acute inflammation (**A**), C-X-C chemokines (**B**), d C-C chemokines (**C**) are shown. Error bars represent standard deviation. Data points indicate individual mice (n=3-5 per group) from one representative experiment that was performed two times. Data were analyzed via one-way ANOVA with Tukey's post-hoc analysis for multiple comparisons. *p<0.05, ***p<0.001.

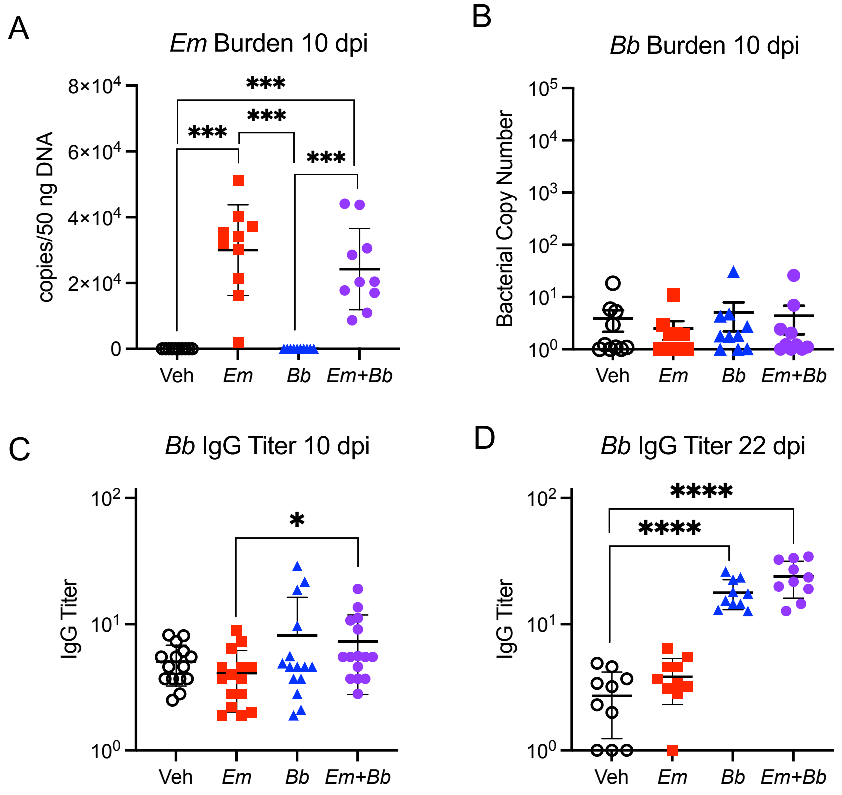

Supplemental Figure 2. Evaluating infection of mice with single and co-infection. B6 mice were inoculated with Vehicle (Veh), *Em (Em)*, *Bb (Bb)*, or both pathogens (*Em*+*Bb*) and euthanized at day 10 or 22 post-inoculation for evaluation of bacterial burden and antibody titers. (**A**) Bacterial burdens in spleen tissue for *Em* as determined by qPCR at 10 dpi. (**B**) *Bb* bacteria were assessed in bladder and not detected at 10 dpi. (**C**) Anti- *Bb* IgG antibodies at day 10 were detected in mice inoculated with *Em* and *Bb*. (**D**) Anti-*Bb* IgG antibodies were detected in both single *Bb* and co-infected animals at day 22 (**D**). Data points represent individual mice pooled from 3 experiments, n=10-15, error bars represent mean and standard deviation. Groups were compared via one-way ANOVA with Tukey's post-hoc multiple comparison. *p<0.05, ***p<0.001, ****p<0.0001.

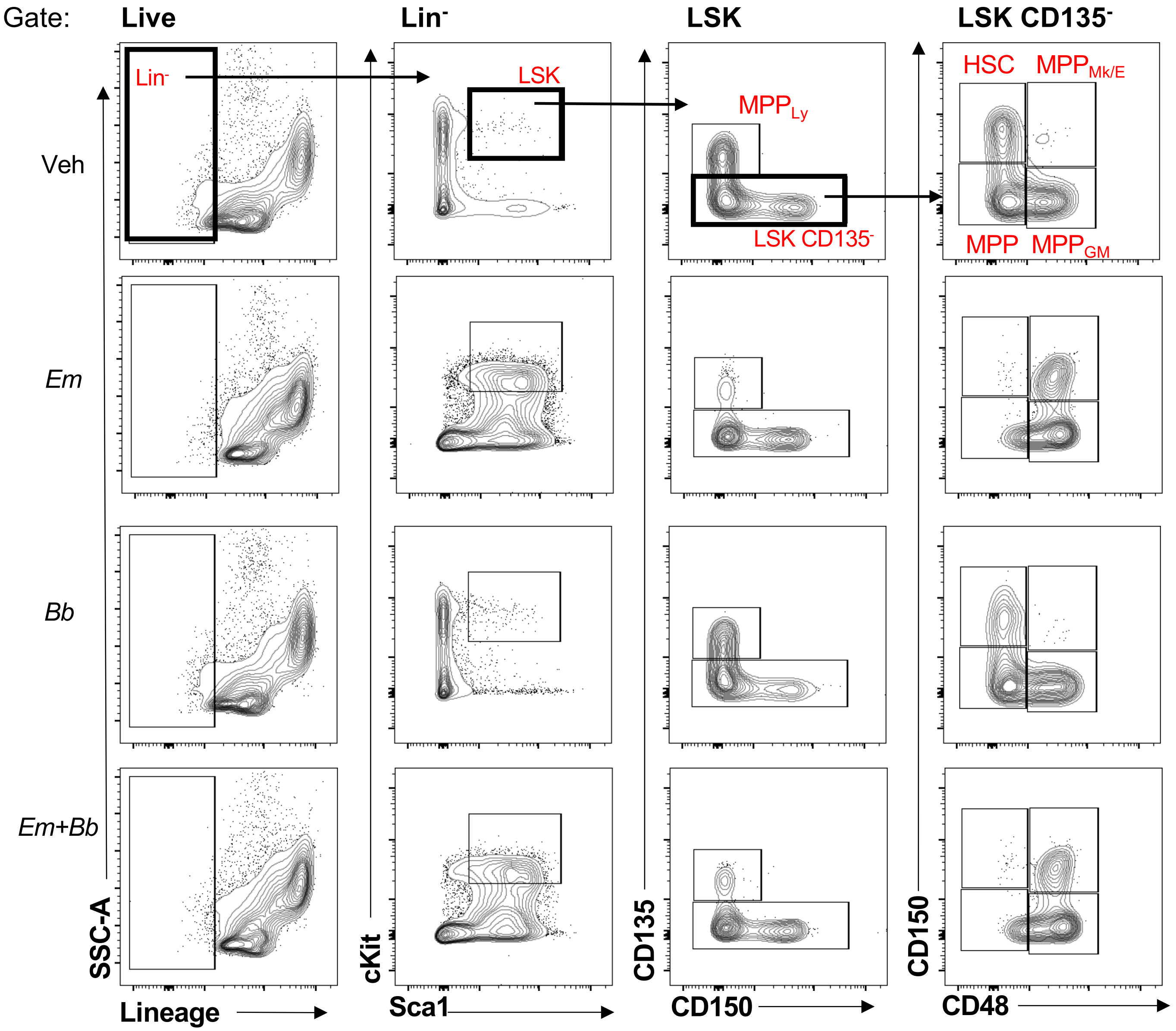

Supplemental Figure 3. Gating strategy to identify bone marrow HSPCs. Representative flow cytometry plots of bone marrow cells from mice inoculated with Vehicle (Veh), *E. muris* (*Em*), *B. burgdorferi (Bb),* or both pathogens *(Em+ Bb)*. Single, live cells were first evaluated for lineage markers (CD3, CD11b, CD45R, Ter-119, GR-1) and the Lin- population was evaluated for expression of cKit and Sca-1 and cells double-positive for both markers are termed LSK. LSK cells were evaluated for expression of CD150 and CD135; lymphoid-biased Multipotent Progenitors (MPP_{Ly}) are $CD150^{-}$ $CD135^{+}$. LSK $CD135^{-}$ cells were then examined for CD150 and CD48 expression and identified as follows: HSCs are LSK $CD135^{-}$ $CD48^{-}$ $CD150^{+}$, MPPs are LSK $CD135^{-}$ $CD150^{-}$ $CD48^{-}$, $MPP_{Mk/E}$ cells are LSK $CD135^{-}$ $CD150^{+}$ $CD48^{+}$, and MPP_{GM} cells are LSK $CD135^{-}$ $CD150^{-}$ $CD48^{+}$.

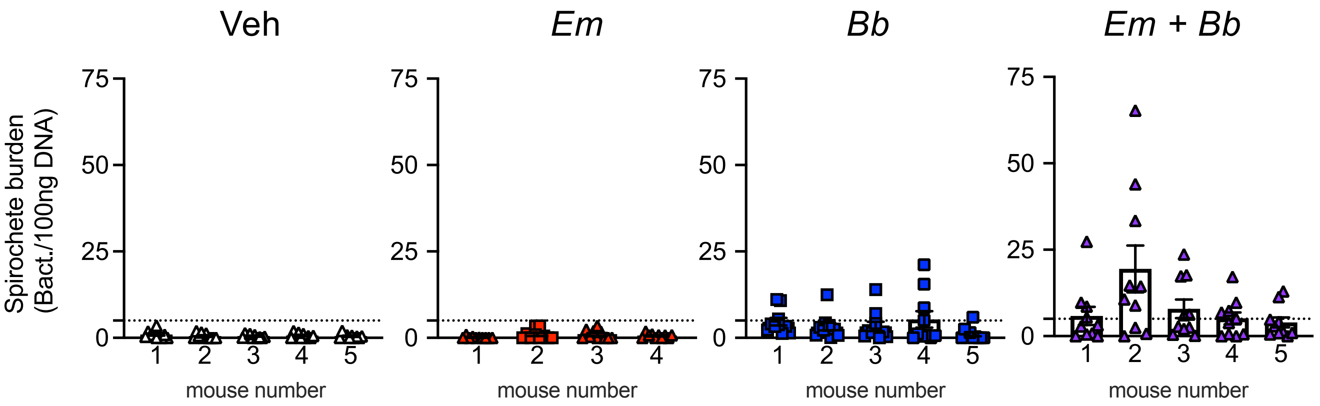

Supplemental Figure 4. Quantification of *Bb* in ticks collected from xenodiagnosis assay mice. C57BL/6 mice were infected with *Em*, *Bb*, or both pathogens as described in previous experiments. Naïve larval ticks were placed on mice as described in the materials and methods, and then collected to evaluate the presence of *Bb*. Data respresent detected Bb burden data from at least 10 ticks collected from individual mice in each infection group.

Table S1: Antibodies for Flow Cytometry

**Antigen**	**Clone**	**Fluor**	**Company (Cat. No.)**
**CD11b**	M1/70	FITC	BioLegend (101206)
**Gr-1**	RB6-8C5	FITC	BioLegend (108406)
**Ter-119**	TER-119	FITC	BioLegend (113206)
**B220**	RA-6B2	FITC	BioLegend (103206)
**CD3e**	17A2	FITC	BioLegend (100204)
**cKit**	2B8	PerCP-Cy5.5	BioLegend (105823)
**CD48**	HM48-1	APC	BioLegend (103411)
**CD150**	TC15-12F12.2	BV711	BioLegend (115941)
**CD135**	AF210	PE	BioLegend (135306)
**Sca-1**	D7	PE-Cy7	BioLegend (108114)
**CD169**	3D6.112	FITC	BioLegend (142406)
**CX3CR1**	SA011F11	PerCP-Cy5.5	BioLegend (149009)
**F4/80**	Cl.AB1	APC	Abcam (AB105080)
**CD11b**	M1/70	Biotin	BioLegend (101204)
**Ly6C**	HK1.4	BV510	BioLegend (128033)
**Ly6G**	1A8	BV605	BioLegend (127639)
**CD3e**	17A2	Biotin	BioLegend (100243)
**CD4**	RM4-5	APC	BioLegend (100412)
**CD8**	53-6.7	BV510	BioLegend (100751)
